# Current clinical trials for craniopharyngiomas: what’s on the horizon?

**DOI:** 10.1007/s11060-024-04899-6

**Published:** 2025-03-05

**Authors:** Nikhil Joshi, Sabine Mueller, Cassie Kline

**Affiliations:** 1 Division of Oncology, Department of Pediatrics, Perelman School of Medicine, Children’s Hospital of Philadelphia, University of Pennsylvania, Philadelphia, PA 19104 USA; 2 Departments of Neurology, Neurological Surgery, and Pediatrics, University of California, San Francisco, CA 94158 USA; 3 Department of Pediatrics, University of Zurich, Zurich, Switzerland

**Keywords:** Craniopharygnioma, Clinical trials, MEK/ERK, BRAFV600E, IL-6, PD-1

## Abstract

Craniopharyngiomas are histologically low-grade tumors in the sellar/suprasellar region that grow close to critical structures including the hypothalamus, pituitary gland, and optic chiasm. Due to this challenging location, many patients face long-term complications including neuroendocrine, neurologic, and visual deficits. As a result, there is interest in developing risk-optimized treatments that minimize damage to adjacent normal tissue and limit chronic complications patients face. In recent years, numerous multi-omic characterizations of craniopharyngioma have identified potential targetable markers of craniopharyngioma. In adamantinomatous craniopharyngioma, numerous clinical trials to explore MEK, PD-1, WNT, and IL-6 inhibition are currently active. In papillary craniopharyngioma, targeting BRAF-V600E and MEK with monotherapy and combined therapies are currently being investigated. Further combining of these therapies with radiation and surgical techniques have potential to change existing treatment paradigms and improve the long-term outcome for patients with craniopharyngioma. With our advanced understanding, clinical investigations that target identified oncogenic drivers of craniopharyngioma should continue to center on therapy options that minimize complications faced by patients with this chronic, high morbidity disease.

## Introduction

**Fig. 1:**
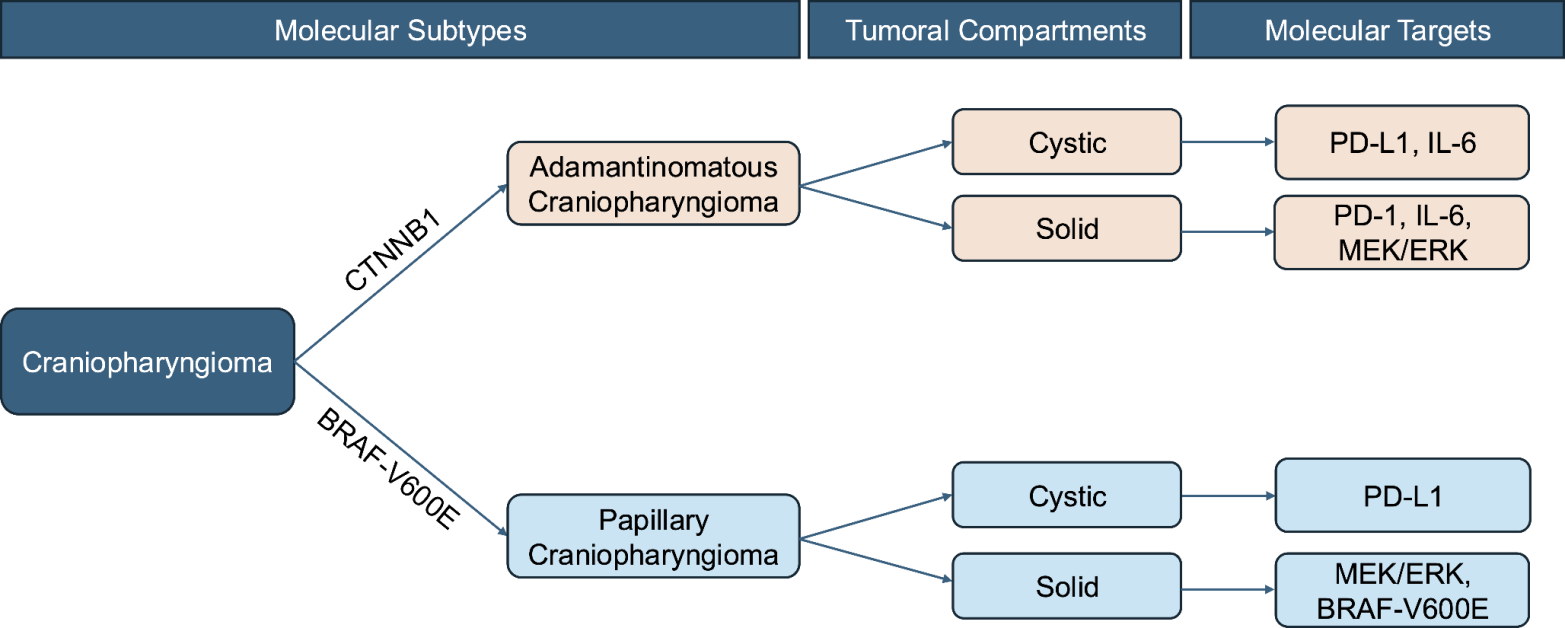
Flowchart depicting subtypes of craniopharyngioma and potential therapeutic vulnerabilities in each AQ3 compartment

Craniopharyngioma is a low histological grade tumor of the sellar/suprasellar region [1]. Overall, craniopharyngioma is seen in 0.5 to 2 individuals per million and accounts for approximately 4% of all pediatric central nervous system tumors [2]. The tumors are classified into two main subtypes based on genomic and histologic features: adamantinomatous craniopharyngioma (ACP), which is commonly characterized by mutations in exon 3 of ß-catenin gene, and papillary craniopharyngioma (PCP), which is commonly characterized by BRAF-V600E gene mutations [3, 4] (Fig. 1). Clinically, ACP is seen in both pediatric and adult patient populations, while PCP is almost exclusively seen in adults [1].

Although craniopharyngioma is considered histologically low-grade, these tumors pose significant challenges to treat and manage due to close localization with critical structures including the hypothalamus, pituitary gland, and optic chiasm. In many cases, these tumors go on to be a chronic disease in affected patients. Due to their anatomically delicate location, patients with craniopharyngioma often face severe endocrine, visual, and neurologic comorbidities that must be considered in the plan of care [5–7]. Hypothalamic syndrome, characterized by intractable weight gain, endocrine abnormalities, memory impairment, and attention deficits with poor impulse control is a serious potential complication seen in children and adults with craniopharyngioma [8]. As a result of the comorbidities related to craniopharyngioma, treatment options often balance considerations of long-term impacts on quality-of-life (QoL) with the goal to minimize risk of tumor recurrence [9]. Many conventional craniopharyngioma therapies that prioritize mitigating recurrence fail to address the high risk of developing tumor- and treatment-related comorbidities [10]. In recent years, advances in neurosurgical approaches and radiotherapy have improved tumor control and decreased risk of therapy comorbidities, but there remain numerous long-term impacts related to tumor and treatment alike, which contribute to worse patient QoL [10]. Even with measures taken to remove or treat the entire tumor, craniopharyngioma recurrence is seen across patients [1, 2, 9]. Recurrence rates though can be markedly different based on extent of surgical resection and radiotherapy, with 12.5% recurrence following gross-total resection, 38% following subtotal resection, and 21% following subtotal resection and radiation [1, 2, 10]. Comorbidities and risks alike contribute to a growing need for innovative therapies that decrease risk of adverse effects and enhance patient outcomes.

In recent years, multi-omic characterization of craniopharyngioma has revealed important insights into the pathology and etiology of this disease, sparking promising clinical trials which treat craniopharyngioma in a patient-specific, risk-optimized manner [11–15]. This review will summarize the current state of treatment in craniopharyngioma and active clinical trials for patients with craniopharyngioma. In addition, we describe characteristic features of ACP and PCP from recent work that will inform future clinical trial development.

## Current treatment landscape

Due to the challenging location of craniopharyngioma to midline brain structures, there is variability and lack of consensus on the most effective and safest approaches for treatment [9]. Treatment for patients can vary greatly based on institutional practice without a true gold-standard approach for craniopharyngioma [9]. Generally, treatment for patients with craniopharyngioma includes a combination of surgical resection and radiotherapy (RT) for residual tumor after resection. However, this is influenced by the extent and characteristics (i.e. solid vs. cystic predominance) of the lesion as well as the age of the patient. Patients with favorably localized craniopharyngioma with no involvement of hypothalamic or optic nerves often undergo gross total resection (GTR) at time of initial diagnosis to minimize possibility of recurrence [16–18]. In patients with unfavorable extent of craniopharyngioma including involvement of surrounding critical structures, there is a growing consensus for subtotal resection (STR) to prevent damage to adjacent brain structures and mitigate chronic impacts on QoL [18, 19]. Numerous studies have demonstrated that STR combined with postsurgical RT decreases the rate of progression and post-operative implications (i.e. hypothalamic obesity) compared with patients that undergo GTR alone [20, 21]. For patients with hypothalamic involvement, retrospective data has shown that STR + RT minimizes treatment-related comorbidities as compared with GTR, but RT still involves risk of pituitary damage, vasculopathy, and radiation-induced malignancy [22–24]. In predominantly cystic lesions, intracystic approaches have been used to achieve local control without impact on surrounding structures. Some examples include the use of interferon-alpha (IFN-a), bleomycin, and phosphorus-32 to target the cystic milieu of ACP [25–27]. Unfortunately, these therapies do not target the solid component of the tumor and there remains risk of neurotoxicity in cases of cyst leakage, including edema, panhypopituitarism, and neurologic injury [12, 28, 29]. With advances in molecular characterization, the next phase of therapy for craniopharyngioma is focused on targeted therapies that minimize risk of neurologic, neuroendocrine, and ophthalmologic injury and limit tumor recurrence by leveraging the distinct features of ACP and PCP [9].

## Recent and ongoing clinical trials in craniopharyngioma

**Table 1:**
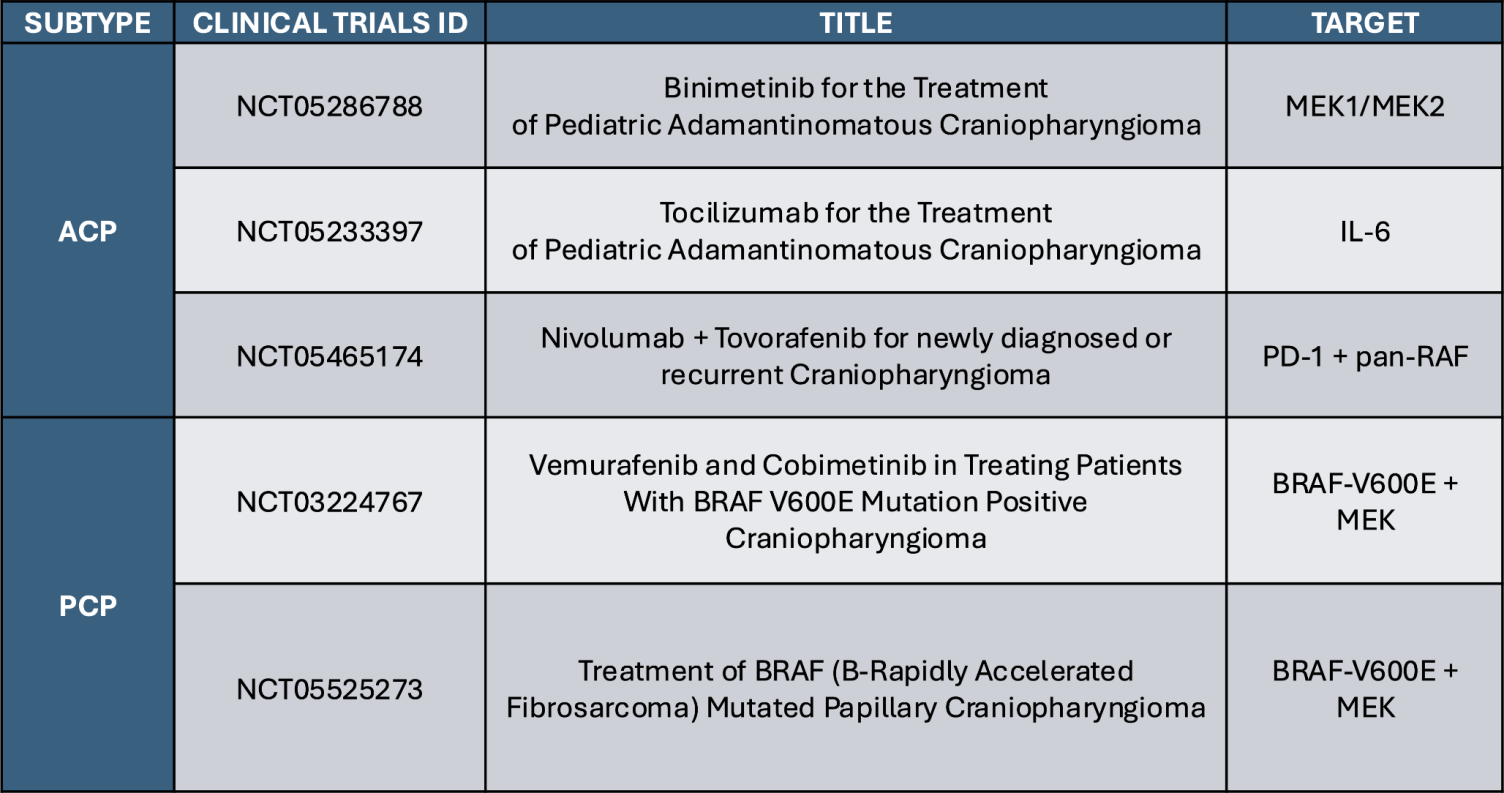
Active clinical trials in craniopharyngioma

In recent years, work in pediatric and adult craniopharyngioma has sought to inform clinical trial development using genomic features of craniopharyngioma in precision-based strategies [12–14]. Numerous clinical studies focused on the molecular and inflammatory components of craniopharyngioma have been developed and are currently underway (Table 1).

### Clinical trials in ACP

In the setting of ACP, both the molecular and inflammatory profile of the tumor make attractive targets of agents under investigation and/or commercially available. Apps et al. characterized a pattern of MAPK/ERK dysregulation and activation in ACP [15]. Petralia et al. similarly characterized that pediatric ACP displayed proteomic profiles with phenotypic similarity to BRAF-V600E mutated low-grade gliomas, further suggesting a potential role for MAPK driven activation in ACP [30]. Based on these characteristics, clinical trials have been developed to shutdown MAPK pathway activation through MEK inhibition, a protein involved in the MAPK pathway. Case studies have demonstrated efficacy of this approach in ACP but there remains limited prospective data on toxicity and clinical outcomes in the context of formal clinical trials [31]. Multiple studies have also characterized the inflammatory environment of ACP. Apps et al. and Hankinson et al. characterized the association of IL-6 expression within a distinct inflammatory phenotype [4, 33, 34]. While expression of this marker is prevalent in the cystic component of ACP, Donson et al. has also shown high proteomic expression of this cytokine in the solid tissue component of ACP suggesting a role for IL-6 in the development of ACP [33]. Recent case studies of tocilizumab, an IL-6 inhibitor, have provided strong rationale for its use as treatment for ACP [34–36]. The PD-1 pathway offers another potentially targetable therapeutic vulnerability as seen in the work of Coy et al. and Petralia et al. [30, 32]. Using the molecular, proteomic, and transcriptomic profiles of ACP, three trials are underway exploring novel targeted strategies – PNOC029 (NCT05465174), CONNECT2108 (NCT05286788), and CONNECT 1905 (NCT05233397) [37–39].

PNOC029 seeks to combine MAPK pathway inhibition with immune checkpoint blockade via PD-1 inhibition in ACP [14, 16, 32, 37]. The trial is open to enrollment for pediatric patients ages 1–39 years with newly diagnosed or recurrent craniopharyngioma. Investigational therapy includes tovorafenib, a type II RAF inhibitor, to target the MAPK pathway in combination with nivolumab to target the PD-1 pathway. Tovorafenib is administered orally every week, while nivolumab is administered intravenously every two weeks. The trial applies a treat-biopsy-treat approach with the goal to characterize the downstream impact of these targeted strategies and inform on mechanisms of response and resistance. The primary endpoint is a composite endpoint, wherein patients must achieve both progression-free survival and maintenance of quality of life at 12 months for this therapy to be deemed a success. To further characterize the QoL and functional outcomes of patients in response to this therapy, PNOC029 is also uniquely collecting follow-up information for three years following therapy start with a specific focus on neuroendocrine and visual outcomes. Importantly, this trial prioritizes these functional outcomes alongside tumor burden as a measure of treatment efficacy. At the same time, this study is collecting robust biospecimens from blood, cyst fluid, tumor tissue, and the microbiome to characterize molecular and immune changes at the patient level, which will inform mechanisms of drug response and guide the next phase of trials for this disease.

Similar to PNOC029, CONNECT2108 aims to target the MAPK pathway in craniopharyngioma [38]. This trial is currently enrolling pediatric patients ages 1–25 years with progressive or recurrent ACP with or without upfront radiation. Patients are treated with binimetinib, an oral, highly-selective MEK1/MEK2 inhibitor, twice a day for up to two years. The primary endpoint of this study is objective response rate, stratified by RT. This study is also exploring biospecimens from blood, cyst fluid and tumor tissue to inform on the biologic effects of this therapy.

CONNECT1905 aims to target the IL-6 pathway in ACP [39]. Building on numerous case studies showing the efficacy of IL-6 use in ACP, this trial is currently enrolling patients ages 1–25 years with progressive or recurrent ACP with or without upfront radiation. Patients are treated with tocilizumab, an IL-6 receptor antagonist, intravenously every two weeks for up to two years. Similar to CONNECT2108, the primary endpoint for this study is objective response rate, stratified by RT, and will also perform collection of blood, cyst fluid, and tumor tissue to characterize patient response to this therapy.

### Clinical trials in PCP

In the setting of PCP, studies have predominantly and successfully explored BRAF inhibitors as a targeted therapy, building on the recent identification that most PCP cases harbor BRAF-V600E mutations [40–43]. Alongside trials exploring BRAF inhibitors alone, BRAF and MEK inhibitors are being explored as combination therapy to reduce the possibility of tumor resistance commonly associated with single-agent BRAF inhibitors. Through this mechanism, trials in the PCP setting have sought to shutdown MAPK pathway activation through BRAF and MEK inhibition. Two trials have combined BRAF/MEK targeting for PCP to explore the benefit of dual inhibition. This includes the “Swecranio” (NCT05525273) and Alliance A071601 (NCT03224767) trials [44, 45]. “Swecranio” seeks to target MAPK pathway activation in BRAF-V600E mutated PCP [44]. The trial is currently enrolling adult patients with non-irradiated, newly diagnosed or recurrent PCP and a confirmed BRAF-V600E mutation. Patients are treated with dabrafenib, a BRAF-V600E inhibitor, alongside trametinib, a MEK inhibitor. Dabrafenib is administered orally twice daily, while trametinib is administered orally once daily. The primary endpoint is to measure reduction in tumor volume, while secondary endpoints include duration of response, progression-free survival and neuroendocrine, visual, and cognitive QoL outcomes. This study will also collect blood throughout treatment to measure changes in circulating BRAF-V600E.

Similar to “Swecranio,” the recently concluded phase 2 Alliance A071601 explored the combination of BRAF and MEK inhibition in PCP [45]. Patients over the age of 18 years with a histologically confirmed diagnosis of a BRAF-V600E driven papillary craniopharyngioma and no upfront radiation were eligible for this study. Enrolled patients received a combination of vemurafenib, a BRAF V600E inhibitor, and cobimetinib, a MEK inhibitor. The primary endpoint was objective response at 4 months. In this trial, investigators saw tumor regression in 94% of the cohort (*n* = 16) with regression proving to be durable over 24 months (24-month PFS: 58%, 24-month OS: 100%) [43]. Most patients (75%) experienced grade 3 or higher adverse events (AEs), with the most common AEs including skin rash and fever, as previously described with these agents [43]. Unfortunately, the long-term neuroendocrine and neurological outcomes patients characteristically struggle with under standard care paradigms have not yet been described in the early stages of follow-up. Longer term efficacy of this combinatorial approach in recurrent PCP also remains unknown. While BRAF-MEK inhibition is not a solution for all PCPs, this targeted therapy suggests a reliable adjuvant therapy for tumor regression that avoids the surrounding tissue damage characteristic of current treatment approaches. However, as with many targeted inhibitors, unknowns in the best use of these agents remain, such as duration of therapy, treatment schedule, development of resistance as well as a rebound after discontinuation of treatment [46].

## Clinical trials on the Horizon

**Table 2:**
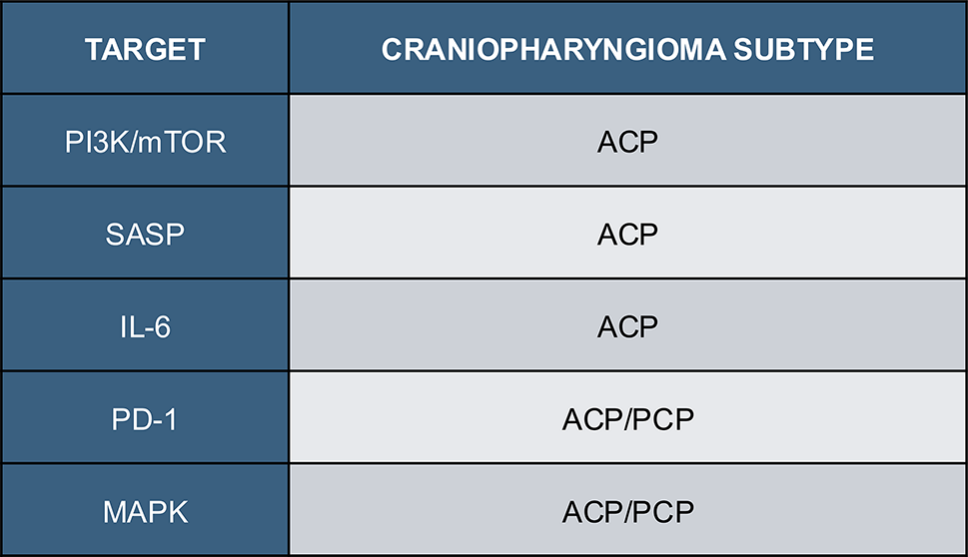
Target considerations for future clinical trials in craniopharyngioma

As therapeutic options for craniopharyngioma are expanded in the future, systemic targeted therapies that leverage the distinct features of craniopharyngioma should continue to be pursued. In the setting of ACP and PCP, recent findings through large single-cell characterizations of patient-derived tissue and preclinical models have provided further insight into cutting edge targets for ACP and PCP clinical trials (Table 2) [13, 14, 47].

### Senescence in ACP

For ACPs, studies have demonstrated the role of senescence in tumorigenesis. In mouse models of ACP, senescent cells induce tumor progression through activation of the senescence-associated secretory phenotype (SASP) [13, 14]. Prince et al. characterized this same phenotype in primary human tissue samples demonstrating a key role for SASP + epithelium in promoting tumor growth and inflammation in ACP. This study and others have demonstrated that SASP secretion by senescent subpopulations within ACP promotes interleukin release (IL1, IL6), MEK/ERK activation and mTOR activation – well characterized tumorigenic pathways seen across ACP biospecimens [13]. This phenotype and similar genomic profiles in the leukemia setting suggest there is potential for senolytic drugs in ACP [48]. In line with these findings, there is a growing interest in developing potential senolytic drugs, such as venetoclax or quercetin, for ACP that can target this senescent, pro-tumoral phenotype in ACP.

### BRAF/MEK in PCP

Building on the findings of the recently completed vemurafenib-cobimetinib trial in PCP, there remain unexplored questions regarding the administration of BRAF/MEK inhibitors in PCP [40]. While initial results from the study showed promising tumor control, there is a growing need to understand the long-term impacts of treatment on neuroendocrine, neurologic and ophthalmological outcomes patients face as well as the durability of tumor-directed response. In line with this, more studies are beginning to explore the optimal timing of BRAF/MEK inhibition in the PCP setting [31]. Encouragingly, the decrease in tumor size and subsequent sparing of normal structures following therapy highlights the potential of this therapy as a possible neo-adjuvant therapy to improve opportunities for surgical and radiation-based treatments [31]. As further studies are designed in PCP, continued optimization of the timing of administration will help determine the integration of BRAF/MEK inhibition for the treatment of PCP.

### PD-1/PD-L1 in ACP/PCP

Additional molecular profiling at a single-cell level and as detailed above, demonstrate the potential application of immunotherapies in ACP and PCP as another promising adjuvant therapy. Jiang et al. showed that in regulatory-T-cell subsets within adult ACP, there is marked expression of exhausted T cell markers including PD-1/PD-L1 and CTLA-4 [14]. On a proteomic level, ACP predominantly expresses PD-L1 on tumor cells in the cyst lining and PD-1 in epithelial subclusters [30]. Within PCP, PD-L1 expression was markedly upregulated in tumor cells surrounding stromal cores. In both ACP and PCP, the expression of PD-1 and PD-L1 co-co-localize with elevated inflammatory signaling throughout tumor clusters. As a result, there is potential for immune modulatory agents to target specific clusters of tumor cells within ACP and PCP and thus, warranting exploration as part of combinatorial strategies (as in PNOC029).

Ultimately, systemic targeted therapies in craniopharyngioma hold much promise and continued characterization of the drivers of ACP and PCP will contextualize the next stage of therapeutic development. Beyond systemic therapies, there is growing interest in alternative radiation therapies and minimally invasive surgical methods in craniopharyngioma. Studies of proton and photon beam radiation have highlighted effective tumor control with reduction in off-target toxicity [49]. Additionally, endoscopic endonasal surgery presents an alternative to transcranial surgical approaches which has the potential for reduced post-operative morbidity [50]. As consideration for novel therapeutic options is expanded in both ACP and PCP settings, there is a need for optimization of administration neoadjuvantly and adjuvantly around radiation and surgery in the care paradigm to minimize complications and recurrence. Amongst all potential therapies, it will be necessary to understand the distinct heterogeneity of craniopharyngioma and the interplay of these pathways that warrant distinct, personalized therapies.

## Considerations for future clinical trial development

The experiences from recent clinical trials in the ACP and PCP settings have provided valuable insight into prospective clinical trial development. Through team-based approaches to clinical trial development there has been rapid acceleration in treatment for patients with craniopharyngioma. Especially due to the rare nature of craniopharyngioma, there is a need for collaborative approaches to ensure the most informative clinical trial designs and collection of robust biospecimens that iteratively inform large scale advances. Through retrospective and prospective clinical, imaging, molecular, and histological data initiatives from the Advancing Treatment in Pediatric Craniopharyngioma (ATPC) Consortium, Pediatric Neuro-Oncology Consortium (PNOC), CONNECT consortium, and Children’s Brain Tumor Network (CBTN), there has been a dramatic increase in the information, preclinical models, and biospecimens available to researchers. Integrated data collection across patient and tumor characteristics and clinical outcomes will further advance our understanding of the complex biology of craniopharyngioma and ideally broaden precision medicine approaches for craniopharyngioma.

The other critical piece to improving outcomes of patients with craniopharyngioma is close surveillance of long-term outcomes, including neuroendocrine, ophthalmologic, and neurologic, and the impact of these outcomes on QoL. Through longitudinal follow-up, we will better understand both the tumor-related effects of prospective therapies and the potential complications they might bring with them. Especially in the setting of craniopharyngioma, where long-term impacts on QoL are valued by patients and families sometimes as much or more than tumor resection, long-term follow-up is necessary to understand the effects of therapies on the QoL [8]. This follow-up, too, can help close the gaps in understanding and addressing the long-term outcomes faced by patients [8]. Clinical trials that specifically focus on patient outcomes across multifocal axes provide valuable models for effectively collecting follow-up on functional and QoL measures. Given the expansive complications patients face, future clinical trials must prioritize measuring these key endpoints as primary and secondary outcomes to place tumor-directed efficacy in perspective of neuroendocrine, visual and neurological outcomes.

## Conclusions

In recent years, advances in the understanding of the biology and molecular features of craniopharyngioma have informed prospective clinical trials in ways not previously seen for ACP and PCP. Targeted systemic therapies are now under investigation, which hopefully translate to therapeutic options with decreased risk to critical structures surrounding the tumor itself and thus, translating to improved QoL. In ACP, targeted therapies inhibiting IL-6, MEK, and PD-1 provide new therapeutic options for patients, but it remains important to identify how these strategies will be used alone, in combination with each other, and alongside surgery and radiation. In PCP, the development of targeted systemic therapies for driver BRAF-V600E mutations and MEK inhibition has shown promising results and should continue to be explored as adjuvant therapy in the care paradigm. In both ACP and PCP, further exploration of immune-modulators and inflammatory inhibition is needed to find risk-optimized therapies for patients. In both settings, clinical trials prospectively exploring the inhibition of these pathways and integration with surgical and radiation therapies will be beneficial to understand how best to improve care. Ultimately, the advances in recent years in craniopharyngioma have shown incredible promise. While several trials have significantly advanced prospects for patients, harnessing the promise of current trials that collect rich data and biospecimens and integrating this data with both historic and prospective data will continue to enhance the survival and functional outcomes for patients.

## Data Availability

No datasets were generated or analysed during the current study.
